# Supplementary figures and images for: Multivariate method for prediction of fumonisins B1 and B2 and zearalenone in Brazilian maize using Near Infrared Spectroscopy (NIR)
Source: PLoS One. 2021 Jan 7;16(1):e0244957. doi: 10.1371/journal.pone.0244957 (PMC7790530; doi:10.1371/journal.pone.0244957)

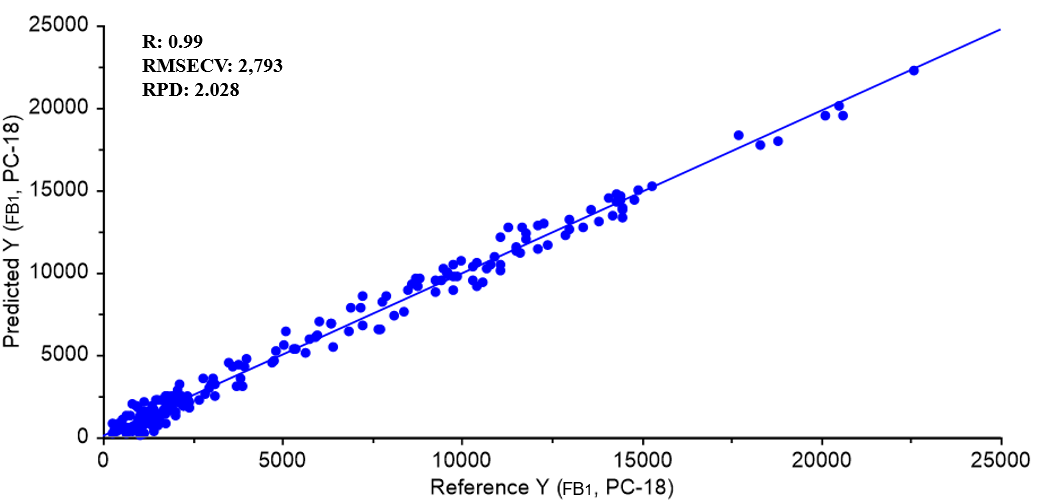

Supplement: S1 Fig — (TIF) [file pone.0244957.s001.tif]

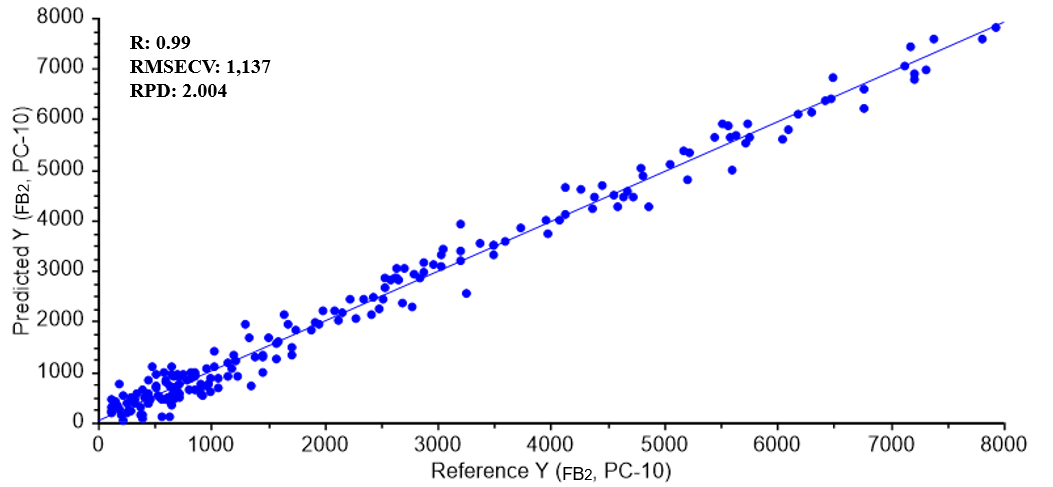

Supplement: S2 Fig — (TIF) [file pone.0244957.s002.tif]

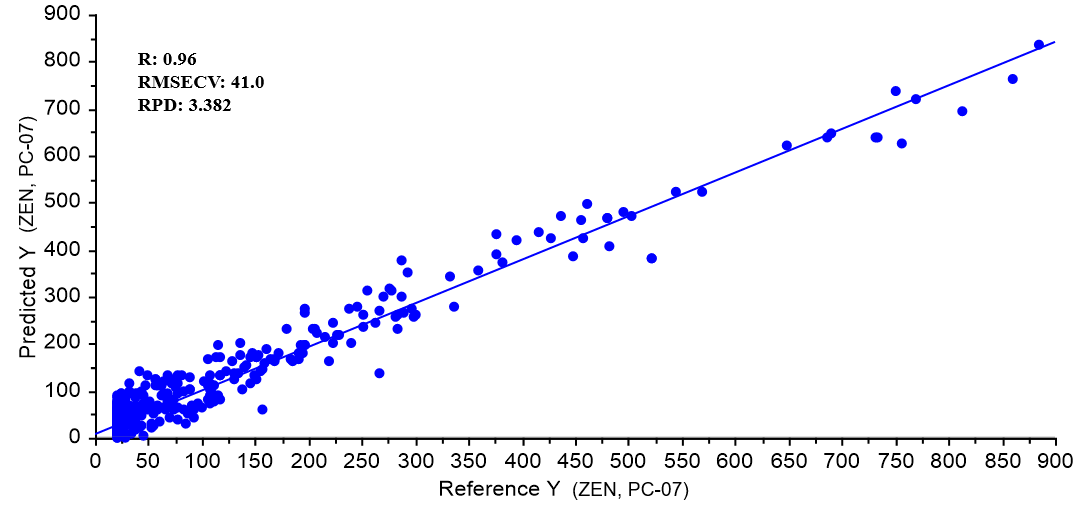

Supplement: S3 Fig — (TIF) [file pone.0244957.s003.tif]
