# Supplementary material for: Multivariate method for prediction of fumonisins B1 and B2 and zearalenone in Brazilian maize using Near Infrared Spectroscopy (NIR)
Source: PLoS One. 2021 Jan 7;16(1):e0244957. doi: 10.1371/journal.pone.0244957 (PMC7790530; doi:10.1371/journal.pone.0244957)
Supplement: Attachment — Submitted filename: Review_PONE-D-20-04995.docx [file pone.0244957.s004.docx]

**PLOS ONE Multivariate method for prediction of fumonisins B1 and B2 in maize using Near Infrared Spectroscopy (NIR) - PONE-D-20-04995**

**General comments**

The manuscript is written very clearly and appropriate methods are used to come to the results. However, it has one major drawback: The manuscript does not present any new information. If I compare the manuscript to that of e.g. Gaspardo et al. (2012, <https://doi.org/10.1016/j.foodchem.2012.06.078>). A similar method is used and I see exactly the same graphs and the descriptions are even very similar E.g. In Gaspardo et al. (2012) it was written: *The data set was organised in a matrix with 143 rows and 926 + 1 columns, combining spectral and chemical data for each sample.* In the current manuscript, it is written: *The calibration set was arranged in a matrix with 236 rows and 4200 + 1 columns, combining spectral and chemical data for each sample*. I can understand that this is indeed the way to deal with this information. But, the main issue is that I cannot really find new findings in this manuscript. You use the same methods for the same toxins in the same matrix. In addition, in 2018, Levasseur-Garcia, presented a clear review on this topic summarizing the toxins and matrices that could be measured with this method (doi: [10.3390/toxins10010038](https://dx.doi.org/10.3390%2Ftoxins10010038)). In addition, the model presented in PONE-D-20-04995 seems to fit the data quiet well, but it is never mentioned which wavelengths or regions in the spectrum are important for toxin quantification.

I suggested a major revision, since I would like to give the authors the chance to significantly improve their work. Now it is merely a copy of what has been done already a lot of times. If they can include e.g. new toxins in a new matrix, or detect mixtures of toxins… or if they can compare several other machine learning technique or new deep learning methods, so that the manuscript had some added value compared to what has been published in the past, I am willing to review this manuscript.

**Specific comments**

L27: *Fumonisins (FBs) are mycotoxins which are major phytopathogens* => Fumonisins are no pathogens they are produced by phytopathogens.

L28: *produced mainly by Fusarium proliferatum and Fusarium moliniforme =>* F. verticillioides is also an important producer of Fumonisins

L35: c*ross validation* => cross-validation

L38: *No significant difference was observed between predicted values using NIR and reference values using Liquid Chromatography Coupled to Tandem Mass Spectrometry (LC-MS/MS) (p > 0.05).* => This is only mentioned in the abstract, I never found it back in the results section, in addition which statistical test was used to test this?

L50: *These 50 mycotoxins are chief phytopathogens of maize* => Toxins are no pathogens

**Fig 5**. What is the difference between the red and the blue line => I suggest calibration and validation, but it is not in the caption which color refers to calibration and which color to validation.

L180: *Several models were developed using different spectral treatments* => Which models? Which spectral treatments?

L182 *as they were considered outliers by the development software* => What is defined as an outlier by the software?

L190 *At the end of this process, the model was reduced to 203 samples for FB1 and 202 samples for FB2, i.e., 33 and 32 samples were excluded, respectively*. => Which samples were excluded. Is there a reason why these samples were excluded? It is always interesting to have an in-depth look at the outliers.
