# Supplementary material for: Multivariate method for prediction of fumonisins B1 and B2 and zearalenone in Brazilian maize using Near Infrared Spectroscopy (NIR)
Source: PLoS One. 2021 Jan 7;16(1):e0244957. doi: 10.1371/journal.pone.0244957 (PMC7790530; doi:10.1371/journal.pone.0244957)
Supplement: Attachment — Submitted filename: Answers_paper.docx [file pone.0244957.s005.docx]

Reviewer #1:

The paper describes the application of the Near Infrared Spectroscopy (NIR) to quantify the level of contamination of total fumonisins in ground maize, and its comparison with well assessed methods such as Liquid Chromatography Coupled Tandem Mass Spectrometry (LC-MS/MS), to verify the suitability of NIR to rapidly analyze a large numbers of maize samples for FBs contamination.

The paper describes experiments well carried out and the conclusions are correct, showing the reliability of this new methods.

However, the paper needs to be deeply revised for the English, that often is very difficult to understand. Also, the way to present data needs to be revised: . I would separate completely Results and Discussion sections in order to have more clear both parts, that in this version are extremely hard to follow.

Finally, minor revisions are reported below.

The manuscript was thoroughly revised following the reviewer's suggestions; furthermore, results and discussion are presented separately.

ABSTRACT

Line 27: the mycotoxins are not phytopathogens. The fungi that produce them are phytopathogens, not the metabolites produced. Eventually, they can be defined phytotoxic compunds.

This information was removed from the manuscript.

Line28: Fusarium moniliforme is not acceptable at all as definition. It is an old nomenclature. Along the whole paper F. moniliforme must be replaced with F. verticillioides

This information was removed from the abstract due to the changes made in the manuscript.

INTRODUCTION

Line 47: Alternaria don’t produce fumonisins B

This information was removed from the manuscript.

Line48: Fusarium moniliforme is not acceptable at all as definition. It is an old nomenclature. Along the whole paper F.moniliforme must be replaced with F. verticillioides

*Fusarium moniliforme* has been replaced by *Fusarium verticillioides* in line 55 and throughout the text.

Line 50: the mycotoxins are not phytopathogens. The fungi that produce them are phytopathogens, not the metabolites produced. Eventually, they can be defined phytotoxic compunds.

This information was removed from the manuscript.

Line 84: replace the word corn with the word maize and replace it along the paper.

The word corn was replaced by maize throughout the manuscript.

REFERENCE

Are the references below reported correctly?

Line 283: 1. RHEEDER J. Fusarium moniliforme and Fumonisins in Corn in Relation to Human Esophageal Cancer in Transkei. 1992;

This reference has been adjusted (line 417).

Line 285 2. BACON CW, NELSON PE. Fumonisin Production in Corn by Toxigenic Strains of 286 Fusarium moniliforme and Fusarium proliferatum. J Food Prot [Internet]. 1994;57(6):514–21. Available from: [https://doi.org/10.4315/0362-028X- 57.6.514](https://doi.org/10.4315/0362-028X-%2057.6.514)

This reference has been adjusted (line 420).

Line 288 3. JACKSON, L.; JABLONSKI J. Fumonisins. In: MAGAN, N.; OLSEN M (Org. ., editor. Mycotoxins in food. Cambridge: Woodhead Publishing; 2004. p. 367–91.

This reference has been adjusted (line 467).

---------------------

Reviewer #2:

The manuscript is written very clearly and appropriate methods are used to come to the results. However, it has one major drawback: The manuscript does not present any new information. If I compare the manuscript to that of e.g. Gaspardo et al. (2012, <https://doi.org/10.1016/j.foodchem.2012.06.078>). A similar method is used and I see exactly the same graphs and the descriptions are even very similar E.g. In Gaspardo et al. (2012) it was written: *The data set was organised in a matrix with 143 rows and 926 + 1 columns, combining spectral and chemical data for each sample.* In the current manuscript, it is written: *The calibration set was arranged in a matrix with 236 rows and 4200 + 1 columns, combining spectral and chemical data for each sample*. I can understand that this is indeed the way to deal with this information. But, the main issue is that I cannot really find new findings in this manuscript. You use the same methods for the same toxins in the same matrix. In addition, in 2018, Levasseur-Garcia, presented a clear review on this topic summarizing the toxins and matrices that could be measured with this method (doi: [10.3390/toxins10010038](https://dx.doi.org/10.3390%2Ftoxins10010038)). In addition, the model presented in PONE-D-20-04995 seems to fit the data quiet well, but it is never mentioned which wavelengths or regions in the spectrum are important for toxin quantification.

I suggested a major revision, since I would like to give the authors the chance to significantly improve their work. Now it is merely a copy of what has been done already a lot of times. If they can include e.g. new toxins in a new matrix, or detect mixtures of toxins… or if they can compare several other machine learning technique or new deep learning methods, so that the manuscript had some added value compared to what has been published in the past, I am willing to review this manuscript.

We are very grateful for the suggestions given to improve the manuscript. We read all the considerations and understood that the paper needed some changes.

Indeed, there are other studies using NIR to predict FBs in maize. However, they were carried out in different countries, with positivity and concentrations that may vary from one region to another. In Brazil, positivity of FBs in maize varies from 90 to 100%, and its inclusion in animal diet is quite high. Furthermore, in the poorest regions of the country, maize is the basis of daily meals, being widely consumed by the population. The use of quick tools, such as NIR, speeds up decision making by the Industry. So, we understand that this study is valid and important from an epidemiological point of view.

We followed your suggestion and included the assessment of another very important mycotoxin: zearalenone. This toxin has been little explored in maize using the NIR technology. Besides, it causes much damage to the reproductive system of farm animals, with significant impacts on production. There are also studies indicating changes in children, associating early puberty with maize-based food contaminated with zearalenone. As I mentioned above, there is a great human consumption of maize-based by-products.

It should also be highlighted that this is the first scientific examination carried out in Brazil using the NIR methodology to predict fumonisins and zearalenone in maize. We used samples from several Brazilian states to compose the database. Many studies are aimed at countries with different climates and frequencies of positivity.

Regarding the investigation of spectral bands, as mentioned in the manuscript, this information remains to be clarified, so we used the entire spectrum. However, work approaching this matter is under development by our research group and it will be promptly published.

**Specific comments**

L27: *Fumonisins (FBs) are mycotoxins which are major phytopathogens* => Fumonisins are no pathogens they are produced by phytopathogens.

This information was removed from the manuscript.

L28: *produced mainly by Fusarium proliferatum and Fusarium moliniforme =>* F. verticillioides is also an important producer of Fumonisins

This information was removed from the abstract due to the changes made in the manuscript.

L35: c*ross validation* => cross-validation

Adjusted as suggested: lines 36, 193 and 242.

L38: *No significant difference was observed between predicted values using NIR and reference values using Liquid Chromatography Coupled to Tandem Mass Spectrometry (LC-MS/MS) (p > 0.05).* => This is only mentioned in the abstract, I never found it back in the results section, in addition which statistical test was used to test this?

All statistical tests used to make comparisons between LC-MS/MS and NIR are mentioned in lines 197 to 200. In the results section, the statistical test and the p value for total fumonisins are mentioned in lines 312-315; the statistical results of zearalenone and the p value are mentioned in lines 325 to 328.

L50: *These 50 mycotoxins are chief phytopathogens of maize* => Toxins are no pathogens

This information was removed from the manuscript.

**Fig 5**. What is the difference between the red and the blue line => I suggest calibration and validation, but it is not in the caption which color refers to calibration and which color to validation.

Fig 5 was adjusted and the captions were inserted: the blue line corresponds to calibration and the red line to validation.

L180: *Several models were developed using different spectral treatments* => Which models? Which spectral treatments?

Several calibration models have been developed using individual and combination mathematical treatments such as smoothing, normalization (mean, maximum and range), baseline offset, multiplicative Scatter Correction (MSC), derivatives, standard normal variate (SNV) and detrending (DT). The model with the best accuracy was selected as the finest one. This information was entered in the results section in lines 242-246.

L182 *as they were considered outliers by the development software* => What is defined as an outlier by the software?

An anomalous sample, also known as an outlier, is an object or, in our case, a sample, which distances itself from the others within the sample set and may not belong to the same population as the majority. Generally, the presence of these samples allows the construction of models with high values of errors and low capacity of prediction, significantly influencing results prediction. Thus, the detection of outliers is of paramount importance and, often, the removal of these samples leads to the construction of more accurate and efficient models, ensuring their predictive quality. A sample can be considered as an outlier due to several reasons: measurement error, noise, extreme samples, deviating products or wrong tabling.

The software offers several regression methods for making models (calibrations). Regression is a generic term used for all methods that try to model and analyze several variables (in our case, 4,200 independent variables, X, and 1 dependent variable, Y) in order to build a relationship between two groups of variables. The adjusted model can then be used only to predict new values.

In regression, there are many ways for a sample to be classified as anomalous. It can be peripheral according to X variables only, or Y variables only, or both. Moreover, it may not be a discrepancy for a separate set of variables, but it can become a discrepancy when considering the relationship (X, Y). We used Partial Least Square (PLS) as a regression method. It is the most suitable for quantitative analysis and the calibrations are generally more robust and can be applied in complex mixtures, as in our case. The fundamental basis of this method is the Principal Component Analysis (PCA), which consists of manipulating the data matrix in order to represent the variations occurring in many variables, through a smaller number of "factors". From this, new systems of axes (called factors, main components, latent variables or even eigenvectors) are constructed to represent the samples, in which the multivariate nature of the data can be visualized in a few dimensions.

The software uses several methodologies to detect outliers, among which are the ones we used: "leverage" and Student residues. Leverages are useful for detecting samples which are far from the center within the space described by the model. Samples with high leverage differ from the average samples; in other words, they are likely outliers. A large leverage also indicates a high influence on the model. Student residues are the concentration residues, which are calculated in cross-validation, that is, poorly modeled samples have high residues.

L190 *At the end of this process, the model was reduced to 203 samples for FB1 and 202 samples for FB2, i.e., 33 and 32 samples were excluded, respectively*. => Which samples were excluded. Is there a reason why these samples were excluded? It is always interesting to have an in-depth look at the outliers.

Yes, it is important to look at these samples carefully, as in some cases it can be a measurement error. The software automatically excludes some samples when creating the model using specific detection tools (for example, leverage and student residue). In this study, the outliers were samples that presented very extreme results and were thus very distant from the population, or they were samples with different characteristics from the others in our database. In such cases, removing these samples is the best alternative. However, the calibrations are dynamic, so from time to time we insert new samples that are considered interesting to make the model more robust and expand the range of sample frequency so that all ranges of concentration and variability are represented.
