# Supplementary material for: Multivariate method for prediction of fumonisins B1 and B2 and zearalenone in Brazilian maize using Near Infrared Spectroscopy (NIR)
Source: PLoS One. 2021 Jan 7;16(1):e0244957. doi: 10.1371/journal.pone.0244957 (PMC7790530; doi:10.1371/journal.pone.0244957)
Supplement: Attachment — Submitted filename: Response to reviewers_03.12.20.docx [file pone.0244957.s006.docx]

**PONE-D-20-04995R1**

**Authors’ responses to reviewers**

Reviewer #1:

I revised the first version of the paper and I think the authors much improved the original paper. Therefore I dont have any further criticism that would not allow the publication of the paper.

- We greatly appreciate your time and effort regarding the revision of our manuscript.

Reviewer #2:

1) In your answer to my first question you mention that the novelty in the article lies in the fact that Many studies are aimed at countries with different climates and frequencies of positivity. Do you mean that samples with ZEN/FB from Brazil will be characterized by another spectrum of samples from another region (if the concentrations are similar)? In my opinion the shift in reflection caused by the presence of a certain toxin at a certain concentration is not highly depended on the region were you took the sample? Please explain in your discussion why it is important that you also do this analysis with samples from Brazil.

- It is important to develop calibrations of more specific databases for each region, since the contamination levels may vary from one place to another; so, samples originating from distinct regions and presenting diverse concentrations are required. In Brazil, for example, the levels of FBs are high, while the levels of ZEN are low. In Europe, in turn, the levels of ZEN and DON are high, while the levels of FBs are low. As suggested, this matter has been further addressed in the discussion section (lines 319 - 325).

2) Another answer was: Regarding the investigation of spectral bands, as mentioned in the manuscript, this information remains to be clarified, so we used the entire spectrum. However, work approaching this matter is under development by our research group and it will be promptly published. Ok you used the entire spectrum to fit the models, but based on the loadings of the PCA analysis and/or coefficients from the PLSR you can easily define characteristic bands for a certain toxin, I want to see this in the article before it can be published, otherwise it does not has an added value for other researchers, the only thing they now know is that you can predict these toxins. But it is important to compare these results with other findings….

- Indeed, we believe this information is relevant for future works, so we included our findings in the discussion section (lines 311-314).

3) In Figure 1 you show the spectrum of the fumonisin samples and of the zearalenone samples. It does not have an added value to divide it into “fumonisin” and “ZEN” samples as the samples you analysed for FB can also contain ZEN and vice versa (and also other toxins)? Did you analyse certain samples for both ZEN and FB, since both toxins often co-occur?

- The samples characterized by the spectra belong to different databases. The figure intended to represent the total amount of these samples and the ranges used. The samples included when building the models were tested in routine laboratory analysis, so not all of them were analyzed for FBs and ZEN simultaneously. Anyhow, this figure was removed from the manuscript.

4) In Figure 5 you show the results of the PCA analysis, however this figure is not very clear. Is it possible to make a 2D biplot and color the dots according to the concentration of ZEN/FB and then also draw the most important loadings so that the reader gains insight into which wavelengths are important for high ZEN or FUM concentrations.

- The image depicted in Figure 5 (now Figure 1) was replaced for a new one showing the most important variables, according to the reviewer’s suggestion.

5) Can you include the models (coefficients of the PLS model) in supplementary data so that they can be tested by the readers?

- This work has been developed along the past 10 years, and several development attempts have been tested. Thus, it is not possible to include the information regarding the coefficients of the PLS model, since it refers to confidential data.

6) I think you can reduce the number of figures, Figures 2-4 can be deleted, Fig. 5 replace it according to the previous comment.

- Figures 2-4 have been removed from the manuscript. Figure 5 was replaced by Figure 1, as suggested by the review.

7) Fig 8-10 can be in the supplementary data.

- Done as suggested (lines 382-389).

8) In the discussion you do not have to refer to figures L365 ZEA => ZEN

- ZEA was replaced for ZEN in lines 76, 290 and 360.
